# Exploring the experiences of stroke survivors, informal caregivers and healthcare providers in Sierra Leone: a qualitative study protocol

**DOI:** 10.1136/bmjopen-2021-051276

**Published:** 2021-12-28

**Authors:** Jessica O'Hara, Melvina Thompson, Gibrilla Deen, Andrew J M Leather, Daniel Youkee, Jurate Wall, Catherine Sackley, Divya Parmar, Christopher McKevitt, NIHR Global Health Research Group at King’s College London, Charles Wolfe

**Affiliations:** 1 King’s Centre for Global Health and Health Partnerships, School of Life Course and Population Sciences, King's College London, London, UK; 2 College of Medicine and Allied Health Sciences, University of Sierra Leone, Freetown, Western Area, Sierra Leone; 3 Faculty of Medicine and Health Sciences, University of Nottingham, Nottingham, UK

**Keywords:** stroke medicine, qualitative research, public health, social medicine

## Abstract

**Introduction:**

Stroke is now the second leading cause of adult death in Sub-Saharan Africa. Developed in dialogue with stroke survivors and caregivers in Sierra Leone, this will be the first study to explore the experience of stroke as well as the perceptions of the barriers and facilitators to accessing stroke care among stroke survivors, informal caregivers and healthcare providers. Findings will inform future stroke research and care in Sierra Leone.

**Methods and analysis:**

A cross-sectional qualitative study employing semistructured interviews that will be audiorecorded, translated, transcribed and coded.

**Setting:**

Freetown, Sierra Leone.

**Participants:**

Interviews with a purposive sample of stroke survivors, informal caregivers and healthcare providers.

**Analysis:**

Interviews will be coded by two authors and inductively analysed using thematic analysis.

**Ethics and dissemination:**

This study has received ethical approval from the Sierra Leone Ethics and Scientific Review Committee (8 December 2020) and the KCL Biomedical & Health Sciences, Dentistry, Medicine and Natural & Mathematical Sciences Research Ethics Subcommittee (reference: HR-20/21-21050). The findings of the study and learning in terms of the process of coproduction and involvement of stroke survivors will be disseminated via peer-reviewed publications, conferences, media and lay reports.

**Strengths and limitations of this study**

- This is the first study to explore experiences of stroke in Sierra Leone.
- Interview guides have been developed with input from stroke survivors and caregivers.
- Interviews will be undertaken by Sierra Leonean researchers trained in qualitative research.
- Findings of the proposed study will contribute to socioculturally informed stroke research and care in Sierra Leone.
- Context-specific learnings may limit the generalisability of the study findings.

## Introduction

Recent trends suggest that Sub-Saharan Africa bears the highest burden of stroke worldwide, with prevalence rates of 14 per 1000, 1-month fatality rates of 40% and age-standardised incidence rates of 316 per 100 000.^1 2^ Strokes in Africa occur at a younger mean age at 57 years compared with 66 years in high-income countries (HICs),^3^ with devastating socioeconomic consequences for stroke survivors, their families and wider communities.^4^ The burden of stroke is projected to increase as African countries undergo epidemiological transitions driven by sociodemographic and lifestyle changes relating to industrialisation.^5 6^

Stroke care provision across Africa is sparce with widespread shortages of qualified healthcare personnel.^6^ Health systems are characterised by geographical and financial inaccessibility, high staff turnover and lack of resources.^7^ A systematic review of stroke care in Africa identifies gaps in the availability of stroke services including CT/MRI scanning machines, stroke units, medical transportation, thrombolysis and rehabilitation services.^6^ Delayed presentation to hospital (median: 1.3 days across Africa)^6^ has been associated with poor awareness of stroke symptoms, delays in transportation and the use of traditional healers.^8^ Studies describing access to outpatient physiotherapy rehabilitation associate low attendance rates with lack of finances and geographic constraints.^9^

A growing body of evidence calls attention to the importance of patient-centred care in improving health processes,^10 11^ outcomes and impact indicators.^12 13^ In HICs, evidence has shed light on how and why patient-centred care works, by exploring the contextual factors underpinning health outcomes.^12 14^

Several authors have identified limitations in the recognition and reporting of context within global health studies, leading to suboptimal operationalisation of interventions.^15^ Qualitative methods are increasingly used to investigate the social processes and phenomena shaping health-seeking behaviours, stroke service provision and intervention implementation.^16^ Owolabi and colleagues^5^ employed qualitative, semistructured interviews and focus groups to better understand the subjective experiences of stroke survivors, informal caregivers and healthcare providers to enable the development of culturally sensitive and system-appropriate interventions in Ghana and Nigeria. Similarly, Jenkins and colleagues^17^ use semistructured interviews and focus groups to explore attitudes, beliefs and practices related to stroke to inform future research and community-based participation and education in Ghana and Nigeria. Findings from the study were then used to adapt the design of a randomised controlled clinical trial of secondary stroke reduction.^18^

Sierra Leone is one of three countries in the Mano River region situated in West-Africa on the Atlantic Ocean. It is one of the least developed countries in the world, with a Human Development Index of 0.419 (184 of 189 countries).^19^ Sierra Leone is recovering from a history of developmental disruptions including a civil war (1991–2002) and an Ebola epidemic (2013–2016). Against the backdrop of economic growth, the country now faces an increasing burden of modifiable stroke risk factors such as diabetes and hypertension.^20 21^ Focusing specifically on stroke, this study seeks to contribute to socioculturally informed research and care in Sierra Leone.

### Research questions

The study will address the following research questions:

- What are stroke survivors’ experiences and perceptions of stroke within the first year of stroke, from onset to rehabilitation?
- What are informal caregivers’ experiences of caring for a stroke survivor within the first year of stroke?
- What are healthcare providers’ experiences and perceptions of stroke care provision?

## Methods and analysis

### Study setting

Connaught Hospital is Sierra Leone’s principal adult referral hospital providing both surgical and medical care in Freetown, the capital city, with an estimated population of 1 million people.^22^ There are no stroke unit services and limited access to CT scanner facilities.^23^

Participants for this study will be purposely sampled from a longitudinal hospital-based stroke register study (SISLE) at Connaught Hospital (National Institute for Health Research Global Health Research Group on Stroke in Sierra Leone, King’s College London (reference: GHRG 17/63/66)). Established in April 2019, the stroke register collects sociodemographic data as well as data on stroke types, care process and outcomes.

### Study design

This is an exploratory qualitative study. Data will be collected through semistructured interviews tailored to each participant group including stroke survivors, informal caregivers and healthcare providers. Interviews will use semistructured guides to facilitate the discussion of sensitive issues, such as social stigma relating to stroke.^24^ It will allow participants to raise issues they feel are important^25^ as well as allow the interviewer to follow-up with questions to facilitate a deeper understanding of the issues raised.

The interview guides have been designed to explore patient, informal caregiver and healthcare provider experiences, and/or perceptions of stroke from onset, to admission, acute care, discharge and life after stroke. The interviewers and participants will have the flexibility to explore emergent themes. Interview guides allow flexibility while still enabling a compare-and-contrast approach when coding and analysing interview transcripts.^25^

### Patient and public involvement

The authors have consulted with the Stroke Association, Sierra Leone (SASL)in developing this qualitative study.SASL area group of SISLE stroke survivors and informal caregivers who meet monthly with healthcare provider representatives and members of the SISLE project. Members of the group first met in July 2019, following an informal engagement event hosted by SISLE. The group serves as a platform for stroke advocacy, peer support and involvement in research. The research questions have been informed by their experiences, preferences and priorities shared during monthly meetings. Patients and caregivers were involved in designing the semistructured interview guides during a workshop held in December 2020. The group piloted the interview guides in January 2021 to assess their relevance and time required to participate in the study. The group will be involved in plans to disseminate the study results to participants and the wider community. Study findings will be presented to the group followed by discussions on implications for improving stroke care and research in Sierra Leone.

### Participant identification and recruitment

The SISLE stroke register team will be responsible for identifying and approaching prospective participants. Stroke survivors will be identified via the SISLE register follow-up pathway and approached during their 3-month or 12-month follow-up survey. Participants will only be approached if they previously agreed to be contacted for further studies during the SISLE register consenting process. Similarly, informal caregivers will be identified and approached during the 3-month or 12-month follow-up surveys of their respective care recipients. Healthcare providers involved in stroke care provision will be approached with permission from the senior management at Connaught Hospital who will provide contact details of staff providing stroke care. Senior management will not be involved in recruitment nor consenting processes to mitigate the risk of pressure to participate. Survivors and caregivers will be approached by the SISLE register team, who are not involved in stroke care provision.

### Inclusion and exclusion criteria

#### Inclusion

Adult stroke survivors (18 years and older) who have the capacity to provide informed consent.

Adult informal caregivers (18 years and older) who are caring for a stroke survivor.

Healthcare providers who have worked at Connaught Hospital for at least 1 year and are involved in stroke care provision.

#### Exclusion

Informal caregivers of deceased register participants will be excluded from the study.

Patient–carer dyads will not be sought to ensure the inclusion of those caring for survivors excluded from the study on the basis of severe cognitive impairment.

Participants will have the option for the interview to take place at their home, at Connaught hospital, or a preferred quiet location. A participant information booklet will be provided before the interview and participants will have the opportunity to ask questions.

### Sample size

Sampling will be purposive to maximise the representation of survivors and caregivers at Connaught hospital. As far as possible, survivors will be recruited to have a mix of respondents based on their clinical status within the stroke register and their gender. Only respondents who have the ability to provide informed consent will be approached. Informal caregivers and healthcare providers will be sampled based on their involvement with the SISLE register study. We will seek to interview an equal number of female and male participants across the three groups.

We aim to conduct up to 50 interviews, but the exact number will be dependent on when we reach saturation, where no new information or themes emerge. It is anticipated that saturation point will be achieved within 50 interviews consisting of:

- Twenty interviews with stroke survivors (10 at their 3-month follow-up and 10 at their 1-year follow-up).
- Twenty informal caregiver interviews (10 caring for a survivor at their 3-month follow-up and 10 caring for a survivor at their 1-year follow-up.
- Ten healthcare provider interviews (including junior and senior physicians, nurses, nursing assistants, physiotherapists and physiotherapy assistants).

### Data collection

Semistructured interviews will be conducted by two interviewers who are native Krio speakers trained in Good Clinical Practice^26^ in a community setting. Interview guides with topics and questions tailored to each participant will be formed and piloted during a 2-day training session with the interviewers. The training session will cover in-depth discussion on the overall study and interview guide (online supplemental appendix 1). During the interviews, interviewers will explain the study objectives and principles of consent. It is anticipated that each interview will last between 45 min and 90 min. Participants will be provided with refreshments, and their transportation costs will be reimbursed. In the event of increased COVID-19 social distancing measures, interviews will be conducted over the phone, using the interview guides, otherwise they will be conducted as per participants’ preference.

10.1136/bmjopen-2021-051276.supp1 Supplementary data

Interviews will be recorded on a standalone audio device. The audio recordings and transcriptions will be held on a password-protected encrypted laptop and backed up on a password-protected cloud server. Interviews will be translated from Krio to English during transcription. The transcriptions will be anonymised, and audio recordings will be securely destroyed after the interview has been transcribed.

Demographic data (age, sex and stroke severity) will be extracted from the stroke register (survivors) for sampling and analysis purposes. Sex, occupation and level of seniority will be collected from formal caregivers.

### Analysis

Thematic analysis using an inductive (data-driven rather than theoretical) approach will be used to identify emergent recurring and/or salient themes in the interview data.^27^ Transcripts will be read and coded by a research assistant, using a qualitative data analysis software NVIVO V.12.^28^ Approximately, 25% of the transcripts will be coded by another member of the research team to ensure validity. The anonymised transcripts will initially be analysed separately to identify themes and relevant codes specific to each participant group. The analysis will then seek to identify common theme consensus, disagreement, and inconsistencies between the three participant groups. Final themes will be reviewed by the wider research team.

### Data protection

All identifiable participant details and consent forms will be kept for 5 years on a password-protected file only accessible to the research team. Audio recordings will be deleted immediately after transcription. Transcriptions will be anonymised. Electronic data will be kept for 15 years on a password-protected secure server. Data management and storage will be subjected to the UK Data Protection Act 1998.

### Ethics and dissemination

Informed, written consent to participate in the study will be obtained from all participants. Illiterate participants or those without motor function will have the option to consent with their thumb print. Ethical approval for this study has been granted by the Sierra Leone Ethics and Scientific Review Committee and the KCL Biomedical & Health Sciences, Dentistry, Medicine and Natural & Mathematical Sciences Research Ethics Subcommittee (reference: HR-20/21-21050).

The information booklets provide clear communication on the voluntary nature of the study. Refusal to participate will not involve a penalty or loss of benefits that the participant is otherwise entitled to. Participants will be informed that they have the right to withdraw from the study at any time by contacting a member of the research team, either in person or by telephone, without having to offer any reason. All contact details are provided on the study information booklets.

The findings of the study and learning in terms of the process of coproduction and involvement of stroke survivors will be disseminated via peer-reviewed publications, conferences, media and lay reports.

The findings of this study will be presented to stakeholders at a workshop where recommendations for future research will be coproduced.

### Declaration of Helsinki

This study complies with the Declaration of Helsinki, adopted by the 18th World Medical Association (WMA) General Assembly, Helsinki, Finland, June 1964 and last revised by the 64th WMA General Assembly, Fortaleza, Brazil, October (2013).

## Discussion

There is limited understanding of the experience of stroke in Sierra Leone. The success of future behavioural interventions to improve health outcomes for stroke patients hinge on their sensitivities to sociocultural contexts that are yet to be explored in global health discourse. The findings of this study will be useful to inform future intervention design in Sierra Leone and contribute to a limited body of literature on the experience of stroke in Sub-Saharan Africa. The usefulness of the data and resulting recommendations are dependent on context and reflective of the views and experiences of stroke survivors, informal caregivers and healthcare providers at Connaught Hospital. Transferability of findings is maximised by the inclusion of multiple stakeholder perspectives and input from the SSSG. A further limitation is the exclusion of stroke survivors unable to provide written, informed consent. This limitation has been mitigated by the opportunity for their caregivers to participate. Future research should consider harder to reach groups such as those who live in rural settings and informal caregivers of patients with stroke who have died.
