# Supplementary material for: Exploring the experiences of stroke survivors, informal caregivers and healthcare providers in Sierra Leone: a qualitative study protocol
Source: BMJ Open. 2021 Dec 28;11(12):e051276. doi: 10.1136/bmjopen-2021-051276 (PMC8719247; doi:10.1136/bmjopen-2021-051276)
Supplement: Supplementary data [file bmjopen-2021-051276supp001.pdf]

Qualitative study Interview Guide – Healthcare providers

Objective: To understand formal healthcare provider's experiences and perceptions of stroke care provision at Connaught hospital.

Section 1: Introduction

a. Please describe your educational background

Probes:

- *What is your formal education level?*

b. Please describe your role at Connaught Hospital

Probes:
- *How long have you worked at Connaught Hospital?*
- *What is your position and responsibilities?*

Section 2: Experience of stroke admission process at Connaught Hospital

a. Please describe a typical admission process for a suspected stroke at Connaught Hospital

Probes:
- *What factors affect the time it takes for stroke patients to present at hospital?*
- *What would improve delays in the time it takes for a stroke patient to arrive at Connaught Hospital?*

b. Do you think the admission process can be improved? If so, how?

Section 3: Experience of stroke care provision at Connaught Hospital

a. Please describe your experience of stroke care provision at Connaught Hospital
b. Please describe any challenges you face in caring for stroke patients at Connaught.

Probes:
- *Do you feel supported in your role?*

c. Please describe the role of informal caregivers at Connaught Hospital.

Section 4: Experience of stroke patient discharge process at

O'Hara J, *et al. BMJ Open* 2021; 11:e051276. doi: 10.1136/bmjopen-2021-051276

Connaught Hospital

d. Please describe a typical discharge process for Stroke patients at Connaught

Probes:

- *Are stroke patients AND caregivers provided with information upon discharge? If so, what kind of information?*
- *Why might stroke survivor's self-discharge early from Connaught?*

Section 5: Experience of providing follow-up stroke care at Connaught Hospital

a. Can you please describe how professionals at Connaught prepare stroke survivors for their recovery at home??

Probes:
- *What recovery services are available at Connaught?*
- *What challenges do you feel stroke survivors face in their recovery?*
- *What would help improve stroke recovery in Sierra Leone?*

b. **How do you think stroke care could be improved in Sierra Leone?**

Probes:

- *What improvements do you think should be made at Connaught Hospital?*
- *What improvements do you think should be made within the community?*
- *What improvements do you think should be made at the population level?*

Section 6: Experience of the SISLE stroke register

a. Please describe your experience of hospital-based registers before the SISLE programme.

b. How has the register changed how stroke care is provided at Connaught Hospital?

Thank you very much for your time and information. Do you have any final comments or questions?

Demographics of the respondent:

- Sex
- Age
- Role/position at the hospital:
